# GABA release by hippocampal astrocytes

**DOI:** 10.3389/fncom.2012.00059

**Published:** 2012-08-17

**Authors:** Karim Le Meur, Juan Mendizabal-Zubiaga, Pedro Grandes, Etienne Audinat

**Affiliations:** ^1^INSERM U603Paris, France; ^2^Université Paris DescartesParis, France; ^3^Centre National de la Recherche Scientifique, Unité Mixte de Recherche 8154Paris, France; ^4^Faculty of Medicine and Dentistry, Department of Neurosciences, University of the Basque Country UPV/EHUBilbao, Spain

**Keywords:** gliotransmission, glia, pyramidal cells, granule cells, glutamate, GABA, taurine

## Abstract

Astrocytes can directly influence neuronal activity through the release of various transmitters acting on membrane receptors expressed by neurons. However, in contrast to glutamate and ATP for instance, the release of GABA (γ-amino-butyric acid) by astrocytes is still poorly documented. Here, we used whole-cell recordings in rat acute brain slices and electron microscopy to test whether hippocampal astrocytes release the inhibitory transmitter GABA. We observed that slow transient inhibitory currents due to the activation of GABA_*A*_ receptors occur spontaneously in principal neurons of the three main hippocampal fields (CA1, CA3, and dentate gyrus). These currents share characteristics with the slow NMDA receptor-mediated currents previously shown to result from astrocytic glutamate release: they occur in the absence of synaptic transmission and have variable kinetics and amplitudes as well as low frequencies. Osmotic pressure reduction, known to enhance transmitter release from astrocytes, similarly increased the frequency of non-synaptic GABA and glutamate currents. Simultaneous occurrence of slow inhibitory and excitatory currents was extremely rare. Yet, electron microscopy examination of immunostained hippocampal sections shows that about 80% of hippocampal astrocytes [positive for glial fibrillary acidic protein (GFAP)] were immunostained for GABA. Our results provide quantitative characteristics of the astrocyte-to-neuron GABAergic signaling. They also suggest that all principal neurons of the hippocampal network are under a dual, excitatory and inhibitory, influence of astrocytes. The relevance of the astrocytic release of GABA, and glutamate, on the physiopathology of the hippocampus remains to be established.

## Introduction

In the central nervous system, astrocytes are in close apposition with neurons and synapses (Schikorski and Stevens, 1997; Grosche et al., 1999; Ventura and Harris, 1999) with which they establish reciprocal functional interactions (Araque et al., 2001; Auld and Robitaille, 2003; Perea et al., 2009). Indeed, beside their ability to sense neuronal activity through the activation of receptors and transporters for neurotransmitters (Marcaggi and Attwell, 2004; Schipke and Kettenmann, 2004), astrocytes can also release chemical transmitters that modulate neuronal excitability and synaptic transmission (Volterra and Meldolesi, 2005; Hamilton and Attwell, 2010; Perea and Araque, 2010; Zorec et al., 2012).

Within the growing list of transmitters released by glia (or gliotransmitters), glutamate, ATP, and more recently D-Serine have received much interest (Volterra and Meldolesi, 2005; Fiacco and McCarthy, 2006; Haydon and Carmignoto, 2006; Oliet and Mothet, 2006). However, other classical transmitters are also released by glial cells and modulate neuronal activity. This has been clearly demonstrated for taurine in the supraoptic nucleus of the hypothalamus where, in response to a decrease of plasma osmolarity, astrocytes release this inhibitory amino acid which then inhibits vasopressin neurons through the activation of glycine receptors (Hussy, 2002 for review). Despite early reports showing the accumulation of γ-amino-butyric acid (GABA) by glial cells (Barres et al., 1990; Gallo et al., 1991; Ochi et al., 1993), the idea that this major inhibitory transmitter of the mammal central nervous system could also be a gliotransmitter has not been so far widely held (Angulo et al., 2008). Recently, however, studies performed in primary cultures (Liu et al., 2000) and in acute brain slices (Barakat and Bordey, 2002; Kozlov et al., 2006; Lee et al., 2010; Jimenez-Gonzalez et al., 2011) demonstrated a release of GABA from glial cells. In particular, astrocytes in the olfactory bulb and in the thalamus inhibit neighboring neurons through the release of GABA which induces slow transient currents mediated by the activation of GABA-A receptors (Kozlov et al., 2006; Jimenez-Gonzalez et al., 2011). These membrane currents have on average slower and more variable kinetics than GABA-A receptor-mediated inhibitory postsynaptic currents (IPSCs). Despite their inhibitory nature, several characteristics of these slow GABAergic currents resemble those of NMDA receptor-mediated currents due to the astrocytic release of glutamate described in other brain areas (Parri et al., 2001; Angulo et al., 2004; Fellin et al., 2004; Perea and Araque, 2005; D'Ascenzo et al., 2007). In particular, slow GABAergic currents can occur simultaneously in neighboring neurons of the olfactory bulb (Kozlov et al., 2006) as this is the case for slow glutamatergic currents of glial origin in the hippocampus (Angulo et al., 2004; Fellin et al., 2004). Interestingly, both excitatory and inhibitory slow currents of glial origin were recorded in granule cells of the olfactory bulb (Kozlov et al., 2006).

The purpose of the present study was to investigate whether inhibitory astrocyte-to-neuron signaling operates in the hippocampus and whether it co-exists with excitatory gliotransmission. Our results show that neurons of all hippocampal fields are under a dual GABAergic and glutamatergic astrocytic influence and that GABA is present in a large proportion, if not all, hippocampal astrocytes.

## Materials and methods

### Slice preparation

All experiments followed European Union and institutional guidelines for the care and use of laboratory animals (Council directive 86/609EEC). Fourteen- to 29-day-old Wistar rats were either anaesthetized with an intraperitoneal (i.p.) injection of a mixture of ketamine (65 mg/kg)/xylazine (14 mg/kg) or humanely killed by cervical dislocation and decapitated. Transverse hippocampal slices (400 μm) were cut in an oxygenated ice-cold solution containing (in mM): 235 Sucrose, 2.5 KCl, 1.25 NaH_2_PO_4_, 0.5 CaCl_2_, 7 MgCl_2_, 20 Glucose, 26 NaHCO_3_, and 5 Pyruvate. The slices were incubated at 34°C for 30 min and then maintained at room temperature for 0.5–4 h in an oxygenated physiological solution containing (in mM): 126 NaCl, 2.5 KCl, 1.25 NaH_2_PO_4_, 2 CaCl_2_, 1 MgCl_2_, 20 Glucose, 26 NaHCO_3_, and 5 Pyruvate. Finally the slices were transferred into a recording chamber perfused at 2.5 ml.min^−1^ with the same solution. Recordings were performed at room temperature. Since lowering osmolarity of the extracellular solution could favor the generation of transient currents (see “Results”) a hypotonic solution containing (in mM): 96 NaCl, 2.5 KCl, 1.25 NaH_2_PO_4_, 2 CaCl_2_, 1 MgCl_2_, 20 Glucose, 26 NaHCO_3_, and 5 Pyruvate has been used in some experiments. In preliminary experiments we substituted chloride for choline to verify that the effect of the hypotonic solution was not due to chloride concentration reduction (*n* = 3, data not shown).

For experiments aiming at inhibiting the vacuolar H^+^-ATPase the slices were incubated for 2.5 h at 34°C in the physiological solution containing 4 μM of the inhibitor bafilomycin A1; the control slices were from the same rat and were incubated in the same conditions but without bafilomycin A1. In all treated slices we verified that no synaptic currents occurred spontaneously or when adding 10 mM potassium chloride to the standard extracellular solution (Angulo et al., 2004; Le Meur et al., 2007).

### Electrophysiology

Pyramidal neurons from the subiculum, the CA1 and CA3 regions, and granule cells from the dentate gyrus were visually identified by means of infrared videomicroscopy. Whole-cell recordings were performed with an intracellular solution containing (in mM): 104 CsGluconate, 10 TEACl, 1 MgCl_2_, 10 HEPES, 10 BAPTA, 5 phosphocreatine, 2 ATP, and 0.3 GTP, the pH was adjusted to 7.3 with CsOH. With this intracellular solution patch pipettes had a resistance of 3–5 MΩ. All potentials were corrected for a junction potential of −10 mV. Most recordings were performed at a holding potential of –30 mV to distinguish excitatory and inhibitory currents on the basis of their polarity. The reversal potential for chloride (E_Cl_) calculated with the Nernst equation was −62 mV.

### Data collection and analysis

Membrane currents were recorded using an Axopatch 200B (Molecular Devices, Sunnyvale, CA, USA) amplifier. They were filtered at 2–5 kH, digitized at 5–20 kHz with a 1322A Digidata (Molecular Devices, Sunnyvale, CA, USA). Series resistance was not compensated but was regularly monitored throughout the experiment using a −1 mV step and recordings showing unstable (>20% increase) series resistance were rejected. Acquisitions and off-line analysis were performed using pClamp9 softwares (Molecular Devices, Sunnyvale, CA, USA). For evaluating statistical differences between two samples Student's *t*-test was performed; when more than two samples were compared an ANOVA test was used, followed by Dunnett's or Tukey tests. Differences were considered to be significant if *p* < 0.05. Values are given as mean ± standard error to the mean (SEM), and n refers to the number of cells unless otherwise stated.

To calculate the probabilities for the occurrence by chance of double events (biphasic currents) resulting from simultaneous slow inward currents (SICs) and slow outward currents (SOCs), we used a Monte Carlo sampling method. We used the average frequencies of SICs and SOCs observed during the 3 min of hypotonic solution applications in 21 DG neurons to estimate the lambda parameters of Poisson laws from which we generated the simulated distributions of SICs and SOCs in 10,000 sample cells, in time windows of 500 ms (corresponding to upper limit of SOC duration), during a period of 3 min. For each sample cell the SICs and SOCs distributions were digitized into 0 (no event) or 1 (at least 1 event) to create a SIC and a SOC vector. Then the SIC and SOC vectors were multiplied (so that the value 1 corresponded to a double event in the resulting vector), and the number of double events was counted for each sample cell. Finally, the sample cells were categorized according to their number of double events, and the frequencies for the observations of different numbers of double events were calculated.

### Drugs

Tetrodotoxin (TTX) was purchased from Latoxan (Valence, France). Strychnine, Gabazine, ATP 2,3-Dioxo-6-nitro-1,2,3,4-tetrahydrobenzo[F]quinoxaline-7-sulfonamine disodium salt (NBQX disodium salt), D-(-)-2-Amino-5-phosphonopentanoic acid (D-AP5), Bafilomycin A1 were purchased from Tocris Cookson (Bristol, UK).

### Double GABA–GFAP (glial fibrillary acidic protein) immunocytochemistry for electron microscopy

Three Wistar rats were deeply anesthetized by i.p. injection of a mixture of Nembutal (5 mg/100 g body weight; Abbott Laboratories Inc., IL, USA) and urethane (130 mg/100 g body weight; Sigma-Aldrich, St. Louis, MO, USA). They were transcardially perfused with PBS (0.1 M, pH 7.4) and then fixed with 500 ml of 2% glutaraldehyde and 1% formaldehyde in PBS. Perfusates were used at 4°C. Tissue blocks were extensively rinsed in 0.1 M PBS (pH 7.4).

Coronal hippocampal vibrosections were cut at 50 μm and collected in 0.1 M PBS (pH 7.4) at RT. Sections were pre-incubated in a blocking solution of 10% bovine serum albumin (BSA), 0.1% sodium azide and 0.02% saponin prepared in Tris-HCl buffered saline (TBS, pH 7.4) for 30 min at RT. A pre-embedding silver-intensified immunogold method and an immunoperoxidase method were used for the co-localization of GABA and GFAP in hippocampal sections. They were incubated in primary rabbit anti-GABA (1:1000; Somogyi and Hodgson, 1985) and mouse anti-GFAP (1:1000; Sigma Chemical Company St. Louis, MO, USA) antibodies both in 10% BSA/TBS containing 0.1% sodium azide and 0.004% saponin on a shaker for 2 days at 4°C. After several washes in 1% BSA/TBS, tissue sections were incubated in a secondary 1.4 nm gold-labeled anti-rabbit IgG (Fab' fragment, 1:100, Nanoprobes Inc., Yaphank, NY, USA) for the detection of GABA, and in a biotinylated anti-mouse IgG (1:200, Vector Laboratories Burlingame, CA, USA) for the detection of GFAP, both in 1% BSA/TBS with 0.004% saponin on a shaker for 4 h at RT. Tissue was washed in 1% BSA/TBS and processed by a conventional avidin–biotin horseradish peroxidase complex method (ABC; Elite, Vector Laboratories Burlingame, CA, USA). Thereafter, tissue was washed again in 1% BSA/TBS overnight at 4°C and postfixed in 1% glutaraldehyde in TBS for 10 min at RT. Following washes in double-distilled water, gold particles were silver-intensified with a HQ Silver kit (Nanoprobes Inc., Yaphank, NY, USA) for about 12 min in the dark and then washed in 0.1 M PB (pH 7.4). Sections were incubated subsequently with 0.05% DAB in 0.1 M PB and 0.01% hydrogen peroxide for 5 min and washed in 0.1 M PB for 2 h at RT.

Stained sections were osmicated (1% OsO4 in 0.1 M PB, pH 7.4, 20 min), dehydrated in graded alcohols to propylene oxide and plastic embedded flat in Epon 812. Eighty nanometer ultrathin sections were collected on mesh nickel grids, stained with uranyl acetate and lead citrate, and examined in a PHILIPS EM2008S electron microscope. Tissue preparations were photographed by using a digital camera coupled to the electron microscope. Figure compositions were scanned at 500 dots per inch (dpi). Labeling and minor adjustments in contrast and brightness were made using Adobe Photoshop (CS, Adobe Systems, San Jose, CA, USA).

### Analysis of the proportion of GABA-immunolabeled GFAP+ profiles in hippocampal CA1 and CA3

Rat hippocampal sections processed for the co-localization of GABA and GFAP with pre-embedding immunocytochemistry were used for semiquantitative analysis. Tissue showing good and reproducible DAB immunoreaction and silver-intensified gold particles were cut at 80 nm. Electron micrographs (10,000–25,000X) were taken from grids (132 μm side) containing DAB immunodeposits; all of them showed a similar DAB labeling intensity indicating that selected areas were at the same depth. Furthermore, to avoid false negatives, only ultrathin sections in the first 1.5 μm from the surface of the tissue block were examined. Positive labeling was considered if at least one immunoparticle was within the cellular profile. Percentages of GABA+ and GABA– astrocytic elements with GAFP were analyzed using a statistical software package (GraphPad Prism 4, GraphPad Software Inc, San Diego, USA).

## Results

### Slow inward and outward currents in principal neurons of the hippocampus

**Figure 1:**
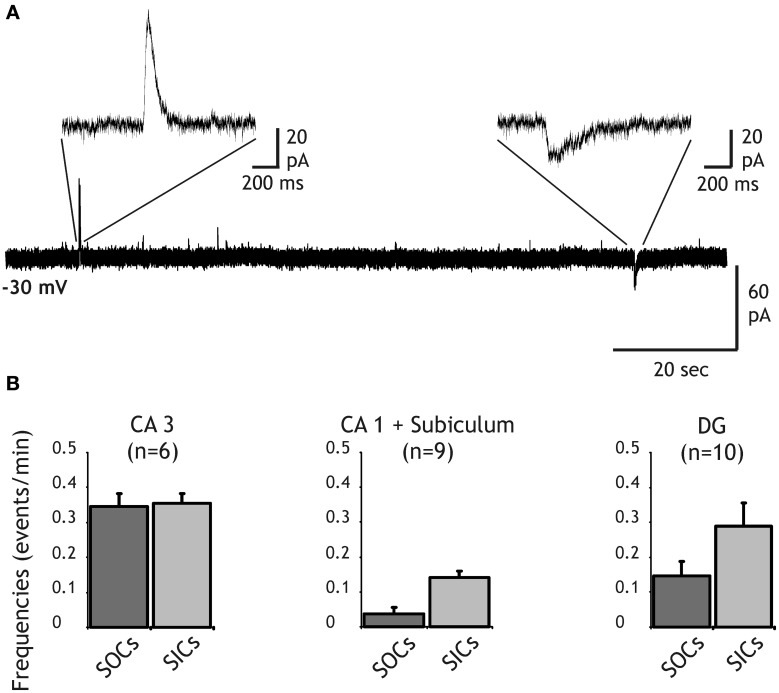
**Distribution of SIC and SOC frequencies in principal neurons of the hippocampus. (A)** Recording from a dentate gyrus granule cell held at a membrane of −30 mV in the presence of TTX (1 μM) and NBQX (10 μM). A slow outward current (SOC) and a slow inward current (SIC) are shown with an expanded time scale. **(B)** Frequency histograms of SOCs (dark bars) and SICS (light bars) recorded in pyramidal cells of CA3 (left), CA1 and the subiculum (middle) and in granule cells of the dentate gyrus (right).

We recorded membrane currents from principal neurons of the hippocampus held at a holding potential of −30 mV to distinguish excitatory and inhibitory currents on the basis of their polarity (see “Materials and Methods”). As previously described (Angulo et al., 2004), in the presence of TTX SICs of glial origin occurred spontaneously at a low frequency in pyramidal neurons of the subiculum and the CA1 regions (Figures 1A,B). A 10–90% rise time >10 ms was used to distinguish SICs from synaptic currents (Angulo et al., 2004). Similar currents were also observed at slightly higher frequencies in CA3 and DG principal neurons (Figures 1A,B). The SIC average frequencies were 0.35 ± 0.03 and 0.29 ± 0.07 events/min in CA3 and in DG, respectively. The 10–90% rise time of SICs was 85.87 ± 10.68 ms and 37.18 ± 4.56 ms in CA3 (*n* = 6 cells, 114 SICs) and in the DG (*n* = 10, 29 SICs), respectively. The amplitude of SICs did not differ significantly in the different regions of the hippocampus and had a mean value of 101.26 ± 10.70 pA (*n* = 259) at a holding potential of −30 mV. Furthermore, bath application of D-AP5 (50–100 μM) or MK801 (50 μM) abolished SICs recorded in CA3 and in DG neurons (*n* = 5; data not shown). Thus, SICs in CA3 and DG principal neurons were generated by the activation of NMDA receptors and were most probably due to the release of glutamate from astrocytes as this is the case for SICs observed in other central neurons (Angulo et al., 2004; Fellin et al., 2004; Perea and Araque, 2005; Kozlov et al., 2006; D'Ascenzo et al., 2007; Pirttimaki and Parri, 2012). It should be noted, however, that values of SIC frequency and amplitude observed here are smaller than those reported in the above mentioned studies. This is most likely due to our recording conditions adjusted to record simultaneously SICs and SOCs, setting smaller driving force for non-selective cationic currents (and for chloride currents) and thus leading to a less favorable signal to noise ratio for the detection of these events.

In addition to SICs, SOCs were also recorded from neurons of all hippocampal regions (Figures 1A,B). A 10–90% rise time >7.5 ms was used to distinguish these slow currents from synaptic currents (see below). Frequency of SOCs was close to that of SICs except in CA1 pyramidal cells in which SOC frequency was about 3 times lower than SIC frequency (0.04 ± 0.02 events/min for SOCs and 0.14 ± 0.02 events/min for SICs; Figure 1B). SOC frequencies in DG and CA3 neurons were 0.15 ± 0.04 events/min and 0.34 ± 0.04 events/min, respectively (Figure 1B). In the olfactory bulb, we showed that SOCs recorded in mitral cells and in granule cells were due to the release of GABA from astrocytes activating neuronal GABA-A receptors (Kozlov et al., 2006). Thus, the occurrence of SOCs in hippocampal neurons suggests that an inhibitory gliotransmitter is also released in the hippocampus.

### Characteristics of SOCs in the hippocampus

**Figure 2:**
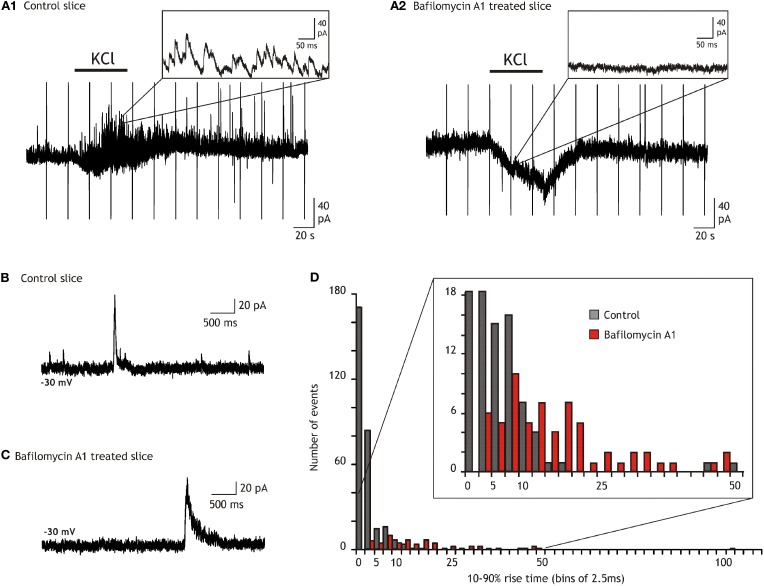
**Comparison of the outward current kinetics recorded in the dentate gyrus of control and bafilomycin A1 treated slices. (A)** Comparison of the synaptic activity and of the effect of bath applied 10 mM KCl on granule cells recorded in a control **(A1)** and in a bafilomycin A1 treated slice **(A2)**. **(B)** Recording from a control slice in the presence of TTX (1 μM), NBQX (20 μM), and AP5 (50 μM). **(C)** Example from a slice treated during 2 h with bafilomycin A1 and recorded in the presence of TTX (1 μM), NBQX (20 μM), and AP5 (50 μM). **(D)** Ten to ninety percentage rise time histograms of the outward currents recorded in control (black) and in bafilomycin A1 treated (red) slices. All currents with amplitude exceeding twice the standard deviation of the noise were considered. Note that the number of currents with rise times faster than 5 ms greatly diminishes in slices treated with bafilomycin A1 while the majority (80%) of the remaining ones, i.e., the SOCs, have rise times slower than 7.5 ms.

**Figure 3:**
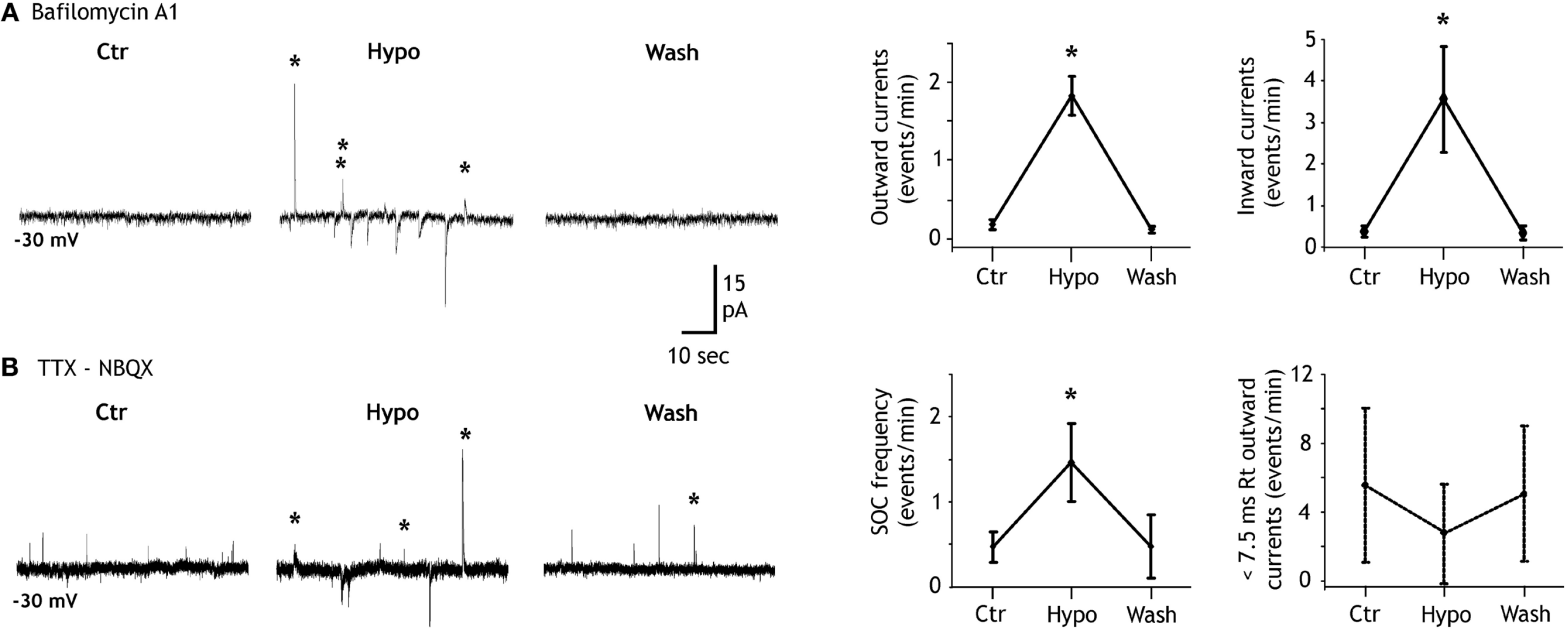
**Hypotonicity increases SIC and SOC frequencies. (A)** Left, example of a granule cell recorded in a bafilomycin A1 treated slice and illustrating the increase of the SOC (labeled with stars) frequency induced by a 20% reduction of the extracellular solution osmoloraty. Note that hypotonicity also increases the frequency of the slow inward currents (SICs). Right, quantification of the effect induced by hypotonic solutions for six granule cells. **(B)** Same as in **A** but in a control slice. Note that the hypotonic solution increases the frequency of SOCs (stars) but not that of synaptic currents (unlabeled outward currents). Right, quantification of the effect induced by hypotonic solutions on the frequency of SOCs (10–90% rise time >7.5 ms; *n* = 8 granule cells; left graph) and on that of miniature IPSCs (10–90% rise time <7.5 ms; same granule cells; right graph).

In a first step to characterize further SOCs in the hippocampus we tested whether they could occur independently of synaptic transmission. We therefore abolished vesicular release by incubating slices for several hours with bafilomycin A1 (4 μM), a blocker of vesicular H^+^-ATPases (Drose and Altendorf, 1997). The abolition of the H^+^ gradient prevents the vesicular accumulation of transmitters and thus synaptic transmission (Zhou et al., 2000; Rossi et al., 2003). In slices treated with bafilomycin A1 and for which we verified that synaptic release was actually abolished (Figures 2A,B; see also “Materials and Methods”) SOCs were still observed in neurons from all hippocampal fields. We concentrated our analysis, however, on CA3 and DG neurons in which SOCs were more frequent than in CA1 cells (see Figure 1). The SOC frequencies in slices treated with bafilomycin A1 were 0.15 ± 0.07 and 0.41 ± 0.28 events/min in the DG (*n* = 6 cells, 64 SOCs) and CA3 (*n* = 9 cells, 57 SOCs), respectively. The SOC amplitude was 71.05 ± 14.99 pA and 46.93 ± 6.93 pA in the DG (*n* = 64 SOCs) and in CA3 (*n* = 57 SOCs), respectively. The 10–90% rise time of SOCs recorded in DG neurons after abolishing vesicular release spanned a large range (3.8–546.5 ms) with a mean of 26.19 ± 8.41 ms (*n* = 64 SOCs) and a distribution such that 83% had a rise time >7.5 ms (Figure 2). The distribution of the 10–90% rise time of SOCs in CA3 was similar, ranging from 5.6 to 162 ms with a mean of 35.70 ± 4.85 (*n* = 57 SOCs). Because some SOCs had a rise time compatible with synaptic currents (Figure 2C), we used a value of 7.5 ms as a threshold to separate SOCs from synaptic currents in all recordings performed in the absence of bafilomycin A1 (e.g., in Figures 1 and 3). Using this criterion, we found that the frequency of SOCs in the DG and CA3 regions was not significantly different in slices treated or not with bafilomycin A1 (see values given above). The distributions of the SOC 10–90% rise times and amplitudes recorded in control slices or in bafilomycin A1 treated slices were not significantly different (not shown). All together these results indicated that SOCs occur spontaneously in the hippocampus and independently of neuronal vesicular release.

We and others have shown that a decrease of the extracellular osmolarity favors the release of transmitter from astrocytes (Pasantes-Morales et al., 1994; Deleuze et al., 1998; Mongin et al., 1999; Takano et al., 2005; Kozlov et al., 2006; Jimenez-Gonzalez et al., 2011) probably through the activation of volume-regulated anion channels(Kimelberg et al., 2006). Accordingly, we observed that the frequency of SOCs recorded in granule cells of the DG was modulated by hypotonic extracellular solutions. In bafilomycin A1 treated slices recorded in the presence of NBQX, lowering the extracellular osmolarity by 15–20% for 1.5–3 min induced an increase of SOC frequency from 0.15 ± 0.07 events/min in control solution to 1.77 ± 0.23 events/min in hypotonic and this effect reverted upon re-introduction of the isotonic extracellular solution in the recording chamber (*n* = 6; Figure 3A). A similar effect of the hypotonic solution was observed on SIC frequency which increased from 0.38 ± 0.13 to 3.55 ± 1.27 events/min (*n* = 6; Figure 3A). SOCs recorded in isotonic or in hypotonic solutions had similar mean amplitude (38.85 ± 12.39 pA, *n* = 18, and 47.02 ± 21.17 pA, *n* = 22, respectively; *p* = 0.7260) and 10–90% rise time (39.53 ± 11.33 ms and 37.17 ± 9.90 ms, respectively; *p* = 0.8825). We also compared the effects of hypotonic solutions on SOCs and inhibitory synaptic activities in control slices that had not been pre-incubated with bafilomycin A1 but were recorded in the presence of TTX and NBQX. In these conditions, hypotonic solutions still increased the SOC frequency but did not changed the frequency of miniature inhibitory synaptic currents (Figure 3B), stressing further the differences between synaptic transmission and the generation of SOCs and SICs.

**Figure 4:**
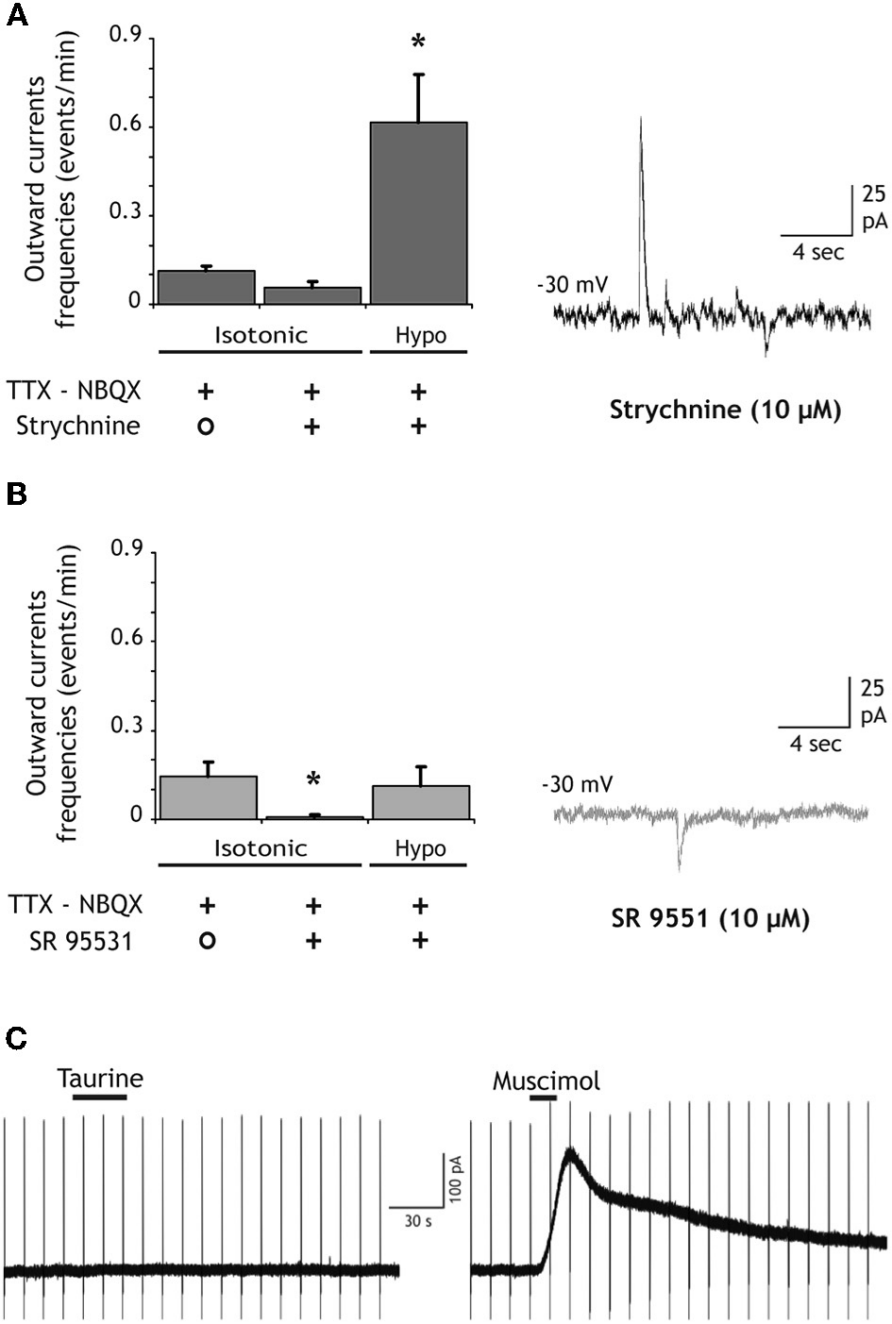
**SOCs are due to the activation of GABA-A receptors. (A)** The competitive antagonist of glycine receptors strychnine (10 μM) does not inhibit SOCs nor does it prevent the increase of SOC frequency induced by hypotonic extracellular solutions (*n* = 9 granule cells). The trace on the right illustrates SOCs and SICs recorded in the presence of strychnine during the application of the hypotonic solution. **(B)** The competitive antagonist of GABA-A receptors SR95531 (GABAzine, 10 μM) strongly reduces the SOC frequency and prevents its increase during the application of hypotonic extracellular solutions (*n* = 11). The trace on the right shows a granule cell recording in the presence of GABAzine during the application of the hypotonic solution. All recordings were performed at −30 mV in the presence of NBQX (10 μM) and TTX (1 μM). **(C)** Taurine does not activate GABA-A receptors of DG granule cells. The left trace shows the absence of response to bath applied taurine (300 μM, 30 s) in a neuron held at −30 mV in the presence of strychnine (10 μM), TTX (1 μM), d-AP5 (40 μM) and NBQX (10 μM). An outward current was induced in the same neuron by muscimol (50 μM, 15 s). Membrane current during applications of 0.2–1 mM taurine varied of average by 2 ± 0.5 pA in DG granule cells (*n* = 7) and by 1.6 ± 2.5 pA in CA3 pyramidal cells (*n* = 4). Vertical deflections in panel **C** correspond to capacitive currents during voltage step commands used to measure membrane and pipette parameters.

SOCs in the olfactory bulb and in the thalamus are due to the activation of GABA-A receptors by GABA released from astrocytes. However, Taurine is another inhibitory aminoacid which can be released by astrocytes (Deleuze et al., 1998; Takano et al., 2005) and could therefore contribute to SOCs by activating glycine receptors expressed by hippocampal neurons (Mori et al., 2002). In granule cells of the DG recorded in slices treated with bafilomycin A1, SOCs (and SICs) were still observed in the presence of the glycine receptor antagonist strychnine (10 μM; Figure 4A). Strychnine did not reduce significantly the frequency of spontaneous SOCs (Figure 4A). Moreover, in the presence of strychnine the application of hypotonic extracellular solutions still increased the SOC frequency (Figure 4A). SOCs recorded in the presence of strychnine had similar mean amplitude (34.07 ± 6.17 pA; *n* = 16 SOCs, *p* = 0.25) and 10–90% rise time (24.98 ± 4.10 ms; *n* = 14 SOCs, *p* = 0.23) than those of SOCs recorded in control conditions. In contrast, bath application of the GABA-A receptor antagonist SR95531 (10 μM) almost abolished all SOCS occurring spontaneously and the application of hypotonic solutions did not induce a significant increase in SOC frequency (Figure 4B). The few remaining SOCs observed in the presence of 10 μM gabazine had smaller amplitude and slower rise times (17.76 ± 6.8 pA and 111.56 ± 19.67 ms, *n* = 5 SOCs) than those observed in control conditions. Moreover, in 4 cells recorded in the presence of 20 μM gabazine, we did not observe any SOCs either in isotonic or hypotonic solutions (not shown).

These results indicate that SOCS in the hippocampus are due to the activation of GABA-A receptors. Yet, they do not exclude that taurine released by astrocytes activates GABA-A receptors. Indeed, Jia and collaborators reported that relatively low concentrations of taurine (10–500 μM) activate extra-synaptic GABA-A receptors of thalamic neurons (Jia et al., 2008). However, in the presence of strychnine (10 μM) to block glycine receptors, bath application of taurine (0.2–1 mM) onto granule cells (*n* = 7) and CA3 pyramidal neurons (*n* = 4) hold at −30 mV failed to induce detectable outward currents while 10–50 μM muscimol readily activated GABA-A receptors in the same recorded cells (Figure 4C). These observations thus strongly suggest that SOCs in hippocampal neurons are due to the release of GABA from astrocytes.

### Relation between SICs and SOCs in the hippocampus

Our results suggest that SICs and SOCs are due to an astrocytic release of glutamate and GABA, respectively. In few cases we observed double currents in which a SIC and a SOC occurred simultaneously (i.e., the second event started before the end of the first one). Over 21 tested neurons, three double events occurred out of 98 SICs and 39 SOCs recorded during the 3 min of hypotonic solution application in these 21 neurons (thus, the average observed frequency of these double events was 0.048 event/min). Using the average frequencies of individual SICs and SOCs in these 21 cells, we performed a Monte Carlo simulation to generate the distributions of SICs and SOCs in 10,000 sample cells and test whether these three double events occurred by chance (see “Materials and Methods”). We obtained only 158 samples with three double events over the 10,000 samples, i.e., the *p*-value for the observation of three double events by chance is less than 0.016, i.e., 3 times lower than the observed experimental value (see above). These results suggest that occurrences of SICs and SOCs are not statistically independent. If true, this would indicate that SICs and SOCs are due to the release of glutamate and GABA, respectively, from the same population of astrocytes or from a heterogeneous population of hippocampal astrocytes having a coordinated activity. Additional experimental evidence is needed to support further this hypothesis.

### GABA immunoreactivity in astrocytes of the hippocampus

**Figure 5:**
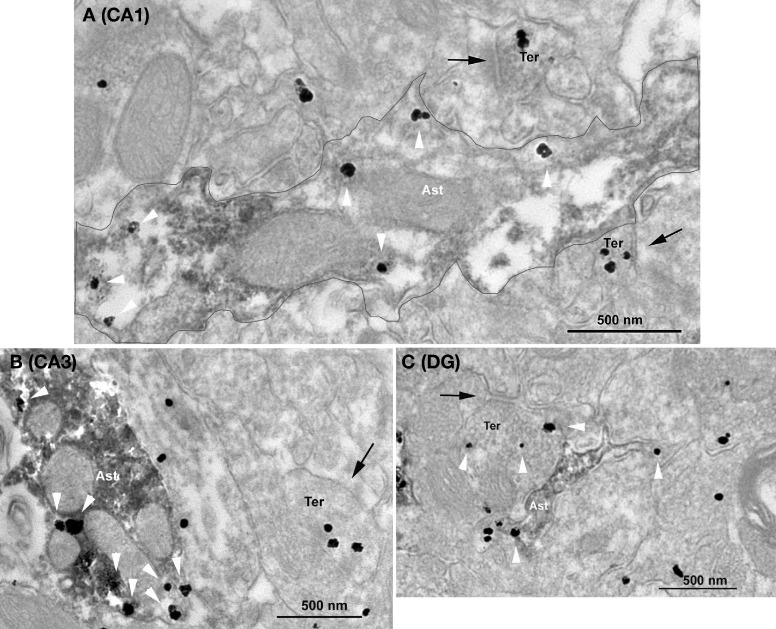
**Ultrastructural immunolocalization of GABA in astrocytes of hippocampal CA1, CA3, and dentate gyrus regions.** Combined pre-embedding immunogold and immunoperoxidase methods for electron microscopy. Astrocytes (GFAP+) and GABA profiles are labeled by DAB and silver-intensified gold particles, respectively. GABA immunoparticles are localized in synaptic terminals (Ter) with symmetric synapses (arrows) as well as in astrocytes (Ast, white arrowheads) in CA1 **(A)** and CA3 **(B)** stratum radiatum and in the dentate gyrus **(C)**. In A, a GAFP+ astrocytic portion containing GABA is delineated.

In order to analyze the presence of GABA of hippocampal astrocytes, we used pre-embedding immunohistochemistry and electron microscopy (see “Materials and Methods”). Astrocytic profiles identified by the presence of GAFP contained GABA immunoparticles in the stratum radiatum of both CA1 and CA3 hippocampal regions (Figure 5) as well as in the DG. Furthermore, the large majority of the analyzed astrocytic elements showed GABA immunolabeling in CA1 (79%, *n* = 53 GFAP positive elements), CA3 (88%, *n* = 34 GFAP positive elements) and in the DG (83%, *n* = 23 GFAP positive elements). These percentages were not statistically different (*p* > 0.05, Pearson's chi-squared test). In all hippocampal regions, small synaptic terminals with pleomorphic synaptic vesicles and forming symmetric synapses with dendritic elements were GABA immunopositive (Figure 5), as expected for the localization of GABA. These results indicate that a large proportion of hippocampal astrocytes contain the inhibitory transmitter GABA.

## Discussion

We and others previously showed that astrocyte release of glutamate triggers SICs in neurons of the thalamus (Parri et al., 2001), in CA1 neurons of the hippocampus (Angulo et al., 2004; Fellin et al., 2004; Perea and Araque, 2005; Nestor et al., 2007), in neurons of the striatum (D'Ascenzo et al., 2007) and of the olfactory bulb (Kozlov et al., 2006). Moreover, astrocytes in the olfactory bulb and in the thalamus also release GABA which activates GABA-A receptors and thereby induces SOCs in neighboring neurons (Kozlov et al., 2006; Jimenez-Gonzalez et al., 2011). Our present results extent these previous observations by showing that SICs and SOCs occur in all principal neurons of the hippocampus, including CA1 and CA3 pyramidal cells and granule cells of the dentate gyrus.

Beside their sensitivity to GABA-A receptor antagonists (see below), SOCs in the hippocampus share several other properties with SOCs previously observed in the olfactory bulb (Kozlov et al., 2006) and in the thalamus (Jimenez-Gonzalez et al., 2011) which distinguish them from inhibitory synaptic currents. In particular, the rise and decay times of SOCs are much slower than those of inhibitory synaptic currents. Moreover, SOCs persist after having inhibited the vesicular H^+^-ATPase, a treatment which readily abolishes synaptic transmission. Finally, lowering extracellular solution osmolarity increases SOC frequency but does not change the frequency of synaptic currents. These observations suggest that a non-vesicular rather than a vesicular mechanism is responsible for the release of transmitter generating SOCs. The dependency of SOC and SIC frequency upon extracellular osmolarity observed in the hippocampus (see Figure 3), in the olfactory bulb (Kozlov et al., 2006), and in the thalamus (Jimenez-Gonzalez et al., 2011) suggests the involvement of volume sensitive chloride channels through which astrocytes can release various amino acids, including glutamate, taurine, and aspartate (Mongin and Kimelberg, 2002, 2005; Takano et al., 2005; Kimelberg et al., 2006). Recently, bestrophyn anion channels (Best 1) have been shown to be responsible for the release of GABA from cerebellar glial cells which generate GABA-A receptor-mediated tonic currents in granule cells (Lee et al., 2010). Unfortunately, blockers of volume-regulated and Best 1 anion channels have non-specific effects on GABA-A receptors mediating SOCs (Kozlov et al., 2006). Thus, further experiments are needed to identify the actual molecular mechanism of release generating SOCs.

Whereas there is little doubt that glutamate is the excitatory gliotransmitter responsible for SICs, both taurine and GABA are potential inhibitory gliotransmitters that could mediate SOCs (Hussy, 2002; Takano et al., 2005; Kozlov et al., 2006). In particular, hippocampal neurons express glycine receptors that can be activated by taurine (Mori et al., 2002). Despite the relatively poor specificity of GABA-A receptor antagonists which can also inhibit glycine receptors (Li and Slaughter, 2007), our results rather support the involvement of GABA-A receptors in mediating SOCs: SOCs were abolished by gabazine at a concentration of 10–20 μM which is 5–10 times lower than the concentration needed to block 50% of the glycine receptor-mediated current produced by 60 μM glycine (Li and Slaughter, 2007). Furthermore, a high concentration of strychnine (i.e., 10 μM) failed to inhibit SOCs. Yet, this observation does not rule out the possibility that SOCs are mediated by an astrocyte release of taurine that would activate extrasynaptic GABA-A receptors (Jia et al., 2008). However, our observation that taurine does not activate GABA-A receptors in DG and CA3 neurons strongly support the idea that GABA is the gliotransmitter responsible for triggering SOCs in these neurons. These results are in keeping with those obtained in the olfactory bulb where the kinetics of SOCs is slower in the presence of nipecotic acid, a blocker of GABA transporters (Kozlov et al., 2006) and where SOCs are also observed in granule cells (Kozlov et al., 2006) that do not respond to taurine application (Belluzzi et al., 2004). Therefore, GABA rather than taurine is probably the inhibitory gliotransmitter mediating SOCs in the hippocampus.

The above conclusion is further supported by the electron microscopy examination of hippocampal tissue immunostained with a specific antibody against GABA (Somogyi and Hodgson, 1985) which demonstrates the presence of GABA in the vast majority of hippocampal GFAP positive glial cells. The existence of a positive correlation between the amount of astrocytic GABA and the amplitude of tonic inhibition has been recently proposed (Yoon et al., 2011). We did not observed major differences in the number of astrocytic processes labeled with GABA antibodies in different hippocampal fields and this contrasts markedly with the fact that the frequency of SOCs was significantly lower in CA1 than in CA3 and in the dentate gyrus. Because SOCs have otherwise similar properties in neurons of the different hippocampal fields, this difference in SOC frequency is thus most likely related to differences in the functional state of astrocytes or in the control of astrocytic GABA release in the different fields rather than due to differences in astrocytic GABA content. A tight regulation of astrocytic release of gliotransmitters is also supported by the extreme low occurrence of simultaneous SICs and SOCs. If these events were merely the result of an uncontrolled leak of transmitters from a cytosolic compartment through anionic channels, one would expect to see mostly double events. Yet, the probability of co-occurrences indicates the existence of some statistical dependency between SICs and SOCs in hypotonic conditions which could be a consequence of an enhanced coordinated activity of the astrocytic network in these conditions.

The physiological roles of GABA and glutamate astrocytic release remain largely unknown. It is noteworthy that both SICs and SOCs can synchronize the activity of neighboring neurons within a given astrocytic domain (Angulo et al., 2004; Fellin et al., 2004; Kozlov et al., 2006; Halassa et al., 2007). Moreover, in the thalamus the incidence of SICs is modulated on the long term by the activity of neuronal glutamate afferents (Pirttimaki et al., 2011). It is thus likely that the impact of SOCs and SICs on neuronal activity, and in particular on the synchronization of neuronal activity, varies according to the overall activity of neuronal networks and according to the physiological and pathological conditions. Along this line, the involvement of SICs in epileptic discharges has been debated (for review Carmignoto and Haydon, 2012) and recently they have been shown to lower the threshold for the generation of ictal discharges in an *in vitro* model of temporal lobe epilepsy (Gomez-Gonzalo et al., 2010). The roles of SOCs in pathological conditions have not yet been evaluated but the fact that they seem to rely on the activation of extrasynaptic receptors (Jimenez-Gonzalez et al., 2011) may also suggests that they play important functions in pathological conditions in which extrasynaptic GABA-A receptors have been implicated (Maguire et al., 2005; Cope et al., 2009). Furthermore, it would be particularly interesting to study the contribution of SOCs in temporal lobe epilepsy in which a shift of chloride equilibrium toward depolarizing potentials contributes to the generation of interictal discharges in the hippocampus (Cohen et al., 2002; Huberfeld et al., 2007). In such conditions, a hyperpolarizing or depolarizing effect of SOCs, inducing small ensemble of neurons to be synchronously inhibited or excited would depend on local regulation of chloride equilibrium at extrasynaptic site.

### Conflict of interest statement

The authors declare that the research was conducted in the absence of any commercial or financial relationships that could be construed as a potential conflict of interest.
